# Supplementary material for: The interactome of intact mitochondria by cross-linking mass spectrometry provides evidence for coexisting respiratory supercomplexes
Source: Mol Cell Proteomics. 2017 Dec 8;17(2):216–32. doi: 10.1074/mcp.RA117.000470 (PMC5795388; doi:10.1074/mcp.RA117.000470)
Supplement: Supplemental Data [file supp_RA117.000470_133922_0_supp_23978_fzffwf.zip › spectra_annotation/mito_spectra_annotation/169-1-6-1-12-1.pdf]

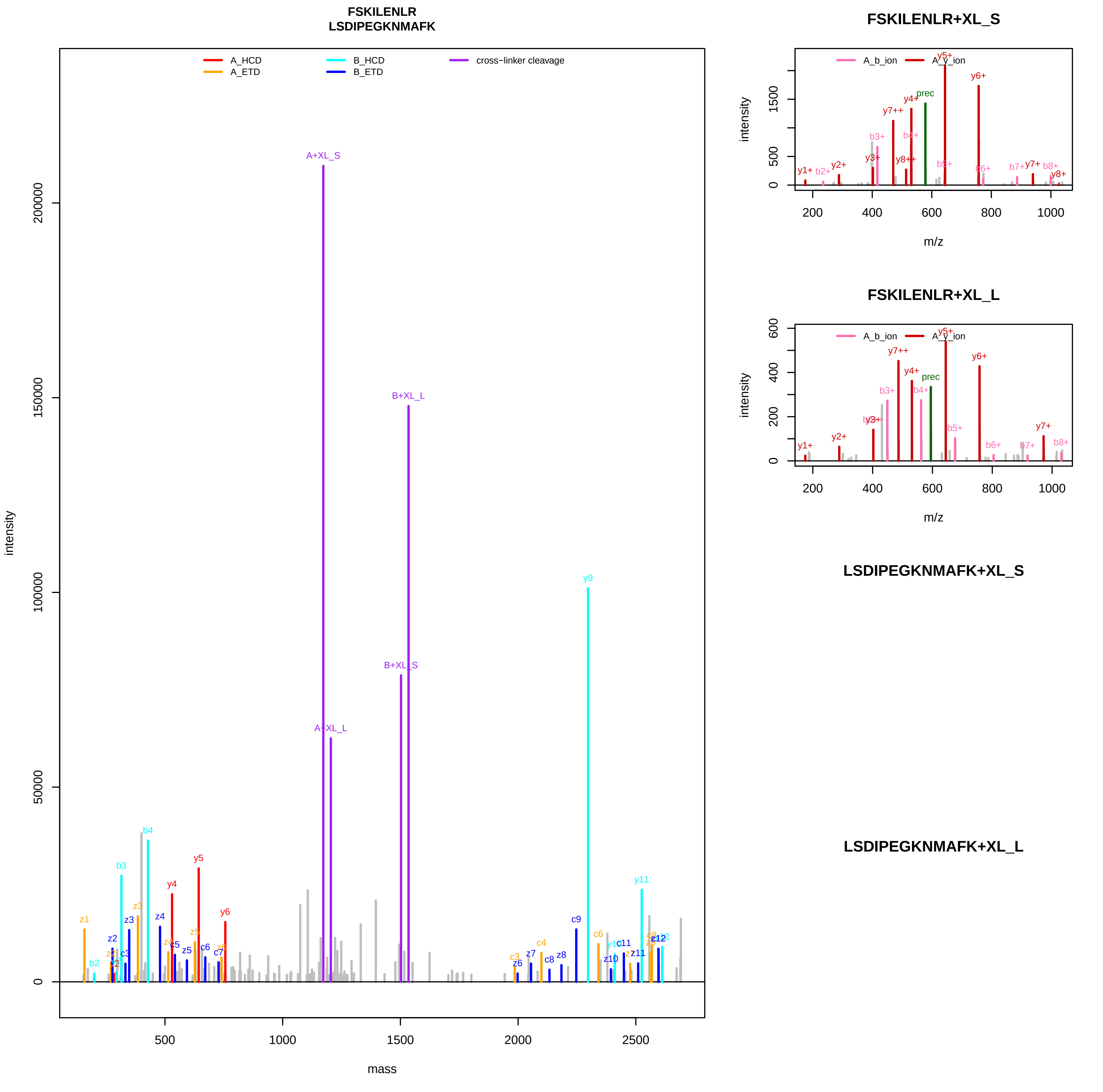
FSKILENLR
LSDIPEGKNMAFK
A_HCD
A_ETD
B_HCD
B_ETD
cross−linker cleavage
A+XL_S
B+XL_L
B+XL_S
A+XL_L
y9
y11
b3
b4
y4
y5
y6
c9
c6
intensity
mass
0
50000
100000
150000
200000
500
1000
1500
2000
2500
FSKILENLR+XL_S
A_b_ion
A_y_ion
prec
y5+
y6+
y4+
y7++
b3+
y3+
y8++
y2+
y1+
b2+
y7+
b8+
y8+
intensity
m/z
0
500
1500
200
400
600
800
1000
FSKILENLR+XL_L
A_b_ion
A_y_ion
prec
y5+
y6+
y4+
y7++
b3+
b4+
b5+
y2+
y1+
b6+
b7+
y7+
b8+
intensity
m/z
0
200
400
600
200
400
600
800
1000
LSDIPEGKNMAFK+XL_S
LSDIPEGKNMAFK+XL_L
